# Innate Immune Regulation: ABHD17 Is Calling NOD2 Back From Duty

**DOI:** 10.1016/j.jcmgh.2025.101493

**Published:** 2025-03-22

**Authors:** Thomas A. Kufer

**Affiliations:** Institute for Nutritional Medicine, Department of Immunology, University of Hohenheim, Stuttgart, Germany

NOD2, a member of the NLR protein family of innate immune receptors, responds towards cytosolic bacterial peptidoglycan fragments and contributes to immune responses. Together with NOD1, this was the first NLR protein to be shown to act as a cytosolic pattern-recognition receptor and to drive activation of the transcription factor nuclear factor kappa B and subsequent release of pro-inflammatory cytokines from host cells.^1^ NOD2 is expressed in a variety of myeloid cells as well as in intestinal epithelial cells. Of clinical relevance, polymorphisms in *NOD2* (*CARD15*) are associated with early onset of Crohn’s disease (CD), a severe inflammatory bowel disorder.^2^ NOD2 activation induces the recruitment of the RIPK2 kinase that subsequently undergoes ubiquitination by the E3 ligase X-linked inhibitor of apoptosis, triggering downstream kinase complexes, resulting in mitogen-activated protein kinase, nuclear factor kappa B activation, and autophagy.^1^ Activation of NOD1/2 and RIPK2 signaling are thereby associated with changes in its subcellular compartmentalization. Twenty years ago, it was first shown that, in epithelial cells, NOD2 is enriched at the plasma membrane and that a CD-associated polymorphism (fs1007) lost this subcellular localization.^3^ Similar results were obtained for NOD1,^4^ and it was shown that membrane localization of both NOD1 and NOD2 correlates with signaling activity.^5^^,^^6^ Upon activation by bacterial membrane vesicles or muramyl dipeptide-coated beads, a redistribution of NOD1/2 also to endosomal membranes is observed, and endosomes are platforms for NOD1/2 activation and signaling in this context.^7^^,^^8^ Concerning the mechanism of membrane recruitment, proteins including FRMPD2^9^ and Erbin-2^10^^,^^11^ were reported as factors that can tether NOD2 at the plasma membrane. In a seminal paper from 2019, S-palmitoylation mediated by the acyltransferase ZDHHC5 was then shown to be a common mechanism for anchoring NOD1 and NOD2 at membranes and to be a prerequisite for signaling.^12^

S-palmitoylation, the covalent linkage of palmitate to cysteine residues of a target protein, is a common mechanism for membrane recruitment and compartmentalization of immune receptors. This reversable post-translational modification is governed by palmitoyl acyltransferases that catalyze the attachment of palmitoyl groups and acyl protein thioesterases that can remove fatty acids from cysteine residues.^13^ Although plasma membrane and endosomal membrane recruitment of NOD2 are needed for signaling, the mechanisms underlying the termination of NOD2 activation remained largely elusive.

Reporting in this issue of *Cellular and Molecular Gastroenterology and Hepatology*, Dixon and co-workers now identified an acyl protein thioesterase involved in the regulation of NOD2 subcellular localization.^14^ They show that isoforms of the α/β-hydrolase domain-containing protein 17 (ABHD17), which also regulates membrane recruitment of PSD95 and of the proto-oncogene N-Ras,^15^ can remove fatty acid chains from NOD2, leading to displacement of NOD2 from membranes and abrogation of signaling. Using human colon cell lines, the authors show that both chemical inhibition and small interfering RNA-mediated depletion of ABHD17 enhances plasma membrane localization of NOD2 and increases muramyl dipeptide-mediated signaling and cytokine expression. Interesting in a clinical context, the authors provide evidence that ABHD17 inhibition can partially rescue membrane localization and signaling competence of select polymorphisms in NOD2 that are associated with CD. It remains to be established if this mechanism can be applied beyond cellular model system and in vivo. Another interesting observation of Dixon et al is that ABHD17 did not affect NOD1 membrane localization; albeit, NOD1 is also S-palmitoylated.^12^ This suggests a more complex regulation of NOD1/2 membrane recruitment, as not all signaling incompetent variants of NOD2 are displaced from the plasma membrane or show impaired RIPK2 interaction.^16^

The results from Dixon et al put ABHD17 proteins at central stage for negative control of NOD2 signaling. They add to our understanding of the spatial aspects of NOD2 activation and highlight the critical interplay between post-translational modifications and receptor localization in maintaining immune homeostasis and responding to microbial threats. Given that CD-associated NOD2 variants show reduced membrane recruitment and lower S-acetylation implies that inhibiting ABHD17 enzymes might offer novel therapeutic strategies for CD intervention.
